# Monitoring Alpha Oscillations and Pupil Dilation across a Performance-Intensity Function

**DOI:** 10.3389/fpsyg.2016.00745

**Published:** 2016-05-24

**Authors:** Catherine M. McMahon, Isabelle Boisvert, Peter de Lissa, Louise Granger, Ronny Ibrahim, Chi Yhun Lo, Kelly Miles, Petra L. Graham

**Affiliations:** ^1^Department of Linguistics, Macquarie University, Sydney, NSWAustralia; ^2^The HEARing CRC, Melbourne, VICAustralia; ^3^Department of Psychology, Macquarie University, Sydney, NSWAustralia; ^4^Department of Statistics, Macquarie University, Sydney, NSWAustralia

**Keywords:** alpha power, pupil dilation, listening effort, listening in noise, speech perception, perceived effort, mental exertion

## Abstract

Listening to degraded speech can be challenging and requires a continuous investment of cognitive resources, which is more challenging for those with hearing loss. However, while alpha power (8–12 Hz) and pupil dilation have been suggested as objective correlates of listening effort, it is not clear whether they assess the same cognitive processes involved, or other sensory and/or neurophysiological mechanisms that are associated with the task. Therefore, the aim of this study is to compare alpha power and pupil dilation during a sentence recognition task in 15 randomized levels of noise (-7 to +7 dB SNR) using highly intelligible (16 channel vocoded) and moderately intelligible (6 channel vocoded) speech. Twenty young normal-hearing adults participated in the study, however, due to extraneous noise, data from only 16 (10 females, 6 males; aged 19–28 years) was used in the Electroencephalography (EEG) analysis and 10 in the pupil analysis. Behavioral testing of perceived effort and speech performance was assessed at 3 fixed SNRs *per* participant and was comparable to sentence recognition performance assessed in the physiological test session for both 16- and 6-channel vocoded sentences. Results showed a significant interaction between channel vocoding for both the alpha power and the pupil size changes. While both measures significantly decreased with more positive SNRs for the 16-channel vocoding, this was not observed with the 6-channel vocoding. The results of this study suggest that these measures may encode different processes involved in speech perception, which show similar trends for highly intelligible speech, but diverge for more spectrally degraded speech. The results to date suggest that these objective correlates of listening effort, and the cognitive processes involved in listening effort, are not yet sufficiently well understood to be used within a clinical setting.

## Introduction

Listening to degraded speech, either in adverse acoustic environments or with hearing loss, is challenging (McCoy et al., 2005; Stenfelt and Rönnberg, 2009), and it is assumed that the increased cognitive load required to understand a conversation is associated with self-reported effort (Lunner et al., 2009; Rudner et al., 2012). Adults with hearing loss report listening to be greatly taxing (Kramer et al., 2006), which may cause increased stress and fatigue (Hétu et al., 1988), contribute to early retirement (Danermark and Gellerstedt, 2004), social withdrawal (Weinstein and Ventry, 1982), and negatively affect relationships (Hétu et al., 1993). Current speech perception tests, which measure performance on a word or sentence recognition task, provide only a gross indication of the activity limitations caused by hearing loss, and do not consider the top–down effects related to increased concentration and attention, as well as effort (Wingfield et al., 2005; Pichora-Fuller and Singh, 2006; Schneider et al., 2010). Therefore, concurrently measuring the cognitive load or listening effort needed to undertake a speech perception task could increase its sensitivity, enabling a more holistic understanding of the challenges faced by adults with hearing loss in communicative settings.

Listening effort, defined as “the mental exertion required to attend to, and understand, an auditory message” (McGarrigle et al., 2014), is influenced by both the clarity of the auditory signal and the cognitive resources available. As hearing loss and cognitive decline are highly associated with age (Salthouse, 2004; Lin et al., 2013), there is a recognized need to understand the contribution of cognition and effort to listening to everyday speech within a clinical environment to better direct rehabilitation strategies towards and/or improve device fitting, particularly for older adults. Certainly it has been shown that greater cognitive resources are required to perceive a speech signal that becomes more degraded and this is more challenging for older adults (Rabbitt, 1991; Rönnberg et al., 2010, 2013). However, importantly, several studies have also highlighted the advantages that individuals with greater cognitive resources have to understand speech in noise (Lunner, 2003), utilize fast signal processing strategies in hearing aids (Lunner and Sundewall-Thorén, 2007), and compensate when mismatches occur between what is heard and the brain’s phonological representations of speech (Avivi-Reich et al., 2014).

Recently, there has been an increased interest in understanding and measuring listening effort, so that future clinical measures may ensue. Many studies have attempted to estimate listening effort, using behavioral, subjective or objective approaches (see McGarrigle et al., 2014 for a review). While subjective measures have high face-validity, they have several inherent limitations; including whether participants are indeed rating perceived effort, or rating their ability to discriminate between different signal-to-noise ratios (SNRs; Rudner et al., 2012). Additionally, subjective measures poorly correlate with other behavioral and objective measures of listening effort (Zekveld et al., 2010; Gosselin and Gagné, 2011; Hornsby, 2013), possibly because these measures relate to specific components of the goal-directed cognitive processes underpinning mental effort (Sarter et al., 2006), therefore each should be investigated. An effective and consistent objective correlate of listening effort has not yet been found (Bernarding et al., 2013), although pupil dilation and oscillations in the alpha frequency band (8–12 Hz) have independently been shown to be associated with changes in speech intelligibility (Obleser et al., 2012; Becker et al., 2013; Zekveld and Kramer, 2014; Petersen et al., 2015) and seem to be sensitive to hearing loss during a speech recognition or digit recall task in noise (Kramer et al., 1997; Zekveld et al., 2011; Petersen et al., 2015). It is, however, not yet known whether these two objective measures assess the same processes, whether sensory (e.g., phonological mapping of degraded speech), cognitive (e.g., cognitive load, inhibition of task irrelevant activity, or working memory), or neurophysiological (e.g., acute stress associated with the investment of attentional resources). These physiological responses may also reflect the extent of brain regions that are recruited to achieve a specific performance (e.g., to increase cognitive processing or provide inhibitory control; see Radulescu et al., 2014). Further, while there is an extensive literature on the neurophysiological mechanisms governing pupil dilation (Laeng et al., 2012), less is understood about those which underpin oscillatory cortical activity or the neuromodulators which influence it (Klimesch et al., 2007).

There appear to be general trends observed between task difficulty and changes in pupil dilation or in alpha power, however, these are not consistent across all studies (see Zekveld and Kramer, 2014; Wöstmann et al., 2015). This may in part depend on the type of task (i.e., listening to randomized or fixed speech tokens), the period when the physiological response is measured (during listening to degraded speech or during the retention period of a memory recall task), or the population characteristics (younger versus older adults, or normal hearing versus those with hearing loss). Alternatively, cognitive load/listening effort may be inherently non-linear and a function of the availability of processing resources coupled with the intentional motivation to allocate such resources to the task (Sarter et al., 2006). That is, when the task is too difficult and the processing demands exceed the available cognitive resources, or when the task is too easy and requires minimal cognitive resources (i.e., is automatic or passive), then effort may not be required or allocated to the task (Granholm et al., 1996; Zekveld and Kramer, 2014). As such, the greatest change in objective measures related to effort may be observable at medium levels of performance, rather than at the extreme ends of performance. Similar non-linear associations between performance and stress (Anderson, 1976) and performance and mental effort have been previously reported (Radulescu et al., 2014).

The current study aims to compare both alpha activity and pupil dilation measured simultaneously over a complete performance-intensity function while listening to sentences with high intelligibility (16-channel vocoded) or moderate intelligibility (6-channel vocoded). Specifically, it aims to identify whether these measures show similar patterns of behavior across the 15 SNRs and with the two levels of vocoding, suggesting that they may encode similar sensory, cognitive or neurophysiological processes involved in listening effort (that currently remain unclear; McGarrigle et al., 2014). A further reason to manipulate both the SNRs and the channel vocoding to degrade speech was to investigate the behavior of these measures on what could be approximated to a simulation of listening with a cochlear implant (Friesen et al., 2001). If these measures are to be applicable in clinical settings, their pattern of behavior should be predictable in a clinical population.

## Materials and Methods

### Participants

Twenty young adults were recruited to participate in this study. Amongst this group, two did not attend all testing sessions. Invalid recordings led to the exclusion of two more participants for the Electroencephalography (EEG) measures and an additional six for the pupil measures. The main reason for excluding the data related to participants looking away from the visual target or closing their eyes when listening became difficult. Participants (10 females, 6 males) were aged from 19 to 28 years (mean = 23 years, *SD* = 2.6). All participants were native Australian English speakers and were right-handed. Participants’ hearing was screened using distortion product otoacoustic emissions. All participants had present emissions bilaterally between 1–4 kHz, which ruled out a moderate or greater hearing loss. All participants reported normal or corrected-to-normal vision. Informed consent was obtained from all participants.

### Speech Perception Material

Recorded Bamford-Kowal-Bench/Australia (BKB/A) sentences spoken by a native Australian-English female were presented as targets in the presence of four-talker babble noise. The sentences and background noise were vocoded by dividing the frequency range from 50 to 6000 Hz into 6 or 16 logarithmically spaced channels. The amplitude envelope was then extracted from each channel and used to modulate the noise with the same frequency band. Each band of noise was then recombined to produce the noise vocoded sentences and background noise. See Shannon et al. (1995) for more information about speech recognition with vocoded material.

### Physiological Measures

Electroencephalography activity and pupil dilation were measured simultaneously during the speech recognition task conducted in a sound-treated and magnetically shielded room. With their forehead resting on an eye-tracker support, participants were asked to maintain their gaze on a small cross presented in the middle of the computer screen. The following presentation protocol was used: 1 s of quiet, variable length of noise (>1 s), sentence in noise, 1 s of noise. Physiological testing was conducted across two sessions: session one used the 16-channel vocoded material and session two used the 6-channel vocoded material. Each session presented 240 target sentences at 65 dB with the noise randomized between 58 and 72 dB (-7 to +7 dB SNR, a total of 15 levels). Pilot data indicated these SNRs provided the full range (0–100%) of speech recognition scores (SRS). The randomization was programmed for sentences of the same BKB/A list to be presented at the same SNR to allow off-line scoring of performance as per the original lists.

After each presentation, a response period of 4 s was given, and indicated by a starting and a finishing tone. Participants were asked to repeat the sentences they heard between the two tone signals, and to guess when unsure. Oral responses were recorded using a voice recorder and video-camera setup directly in front of them, to allow more accurate marking of their responses at a later time. The sentence recognition in noise task was scored at a word level (using the standard BKB/A scoring criteria) and performance was scored for each SNR condition.

#### EEG

A soft-cap was used to facilitate the spatial separation of the electrodes. EEG data were recorded from 32 Ag-AgCl sintered electrodes using the 10–20 montage with a Synamps II amplifier. The ground electrode was located between the Fz and FPz electrodes. Electrode impedances were kept below 5 kΩ. Ocular movement was recorded with bipolar electrodes placed at the outer canthi, and above and below the left eye. Data was recorded at a sampling rate of 1000 Hz, an online band-pass filter of 0.01 to 100 Hz, and a notch filter at 50 Hz.

**FIGURE 1:**
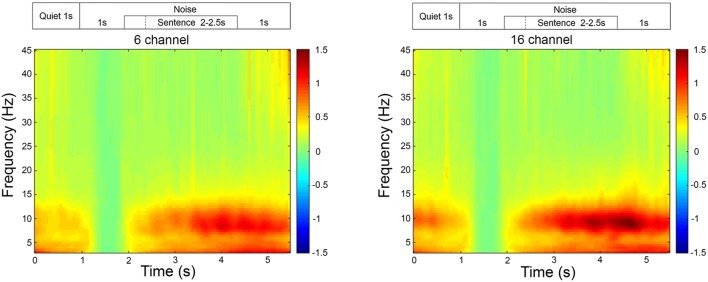
**Time-frequency representation of the EEG activity averaged across all participants, in the frontal and parietal region, for 16- and 6-channel vocoding.** The time-frequency representations are relative to the activity occurring during the 1 s of noise beginning at the 1 s time-point. On this graph, all sentences finished at the 4.5 s time-point.

Post-acquisition, all cortical recordings were analyzed using Fieldtrip, an analysis toolbox in MATLAB developed by Oostenveld et al. (2011). The raw EEG data were first epoched between -2 and 6 s relative to the stimulus onset at 0 s which were then re-referenced to the combined mastoids. The re-referenced epochs were then bandpass filtered with the cut-off frequencies of 0.5 to 45 Hz. Eyeblink artifacts were rejected by transforming the sensor space data into independent components space data using independent component analysis (‘runica’). The eyeblink artifacts were visually inspected and rejected by transforming the components data back into sensor space by excluding the identified eyeblink component(s). Movement related artifacts and noisy trials were rejected by visual inspections. The accepted trials were bandpass filtered again with cut-off frequencies between 8 and 12 Hz to extract alpha oscillations. Alpha band activity was extracted from the parietal electrodes (P3, P4, and Pz) during the encoding period (1 s duration finishing 200 ms before the end of the sentence) and was subtracted from the baseline in noise (300–800 ms after the noise onset) on a trial by trial basis, then averaged to obtain mean alpha power for each SNR. As no significant time-frequency electrode clusters were identified across the scalp during the sentence processing time period, alpha power in the parietal region was used in the current study. A time-frequency representation of the average EEG data collapsed across all of the signal-to-noise levels (**Figure 1**) illustrated the increased activity occurring in the alpha frequency-band averaged during the sentence presentations for both 16-channel and 6-channel noise vocoded sentences.

#### Pupillometry

Pupil size was measured with a monocular (right eye) Eyelink 1000 eye-tracker sampling at 1000 Hz. Single-trial pupil data was processed through Dataviewer software (version 1.11.1), and compiled into single-trial pupil-diameter waveforms (0 s baseline to 6 s) for further oﬄine processing and analyses performed using MATLAB. Data were smoothed using a 5-points moving average.

Blinks were identified in each trial as pupil sample sizes that were smaller than three standard deviations below the mean pupil diameter. Trials where more than 15% of the trial samples were detected as in a blink (which also occurred when the participants were looking away from target) were rejected. In accepted trials, samples within blinks were interpolated from between 66 ms preceding the onset of a blink to 132 ms following the end of a blink. Accepted trials were averaged to form condition-specific pupil size waveforms to represent change of pupil dilation across the trial. For each participant a threshold of 135 or more accepted trials in both the 6- and the 16- channel blocks had to be met to not be excluded, so that a meaningful condition average may be formed. The average of accepted trials for each participant was 193, or 13 trials per SNR.

**FIGURE 2:**
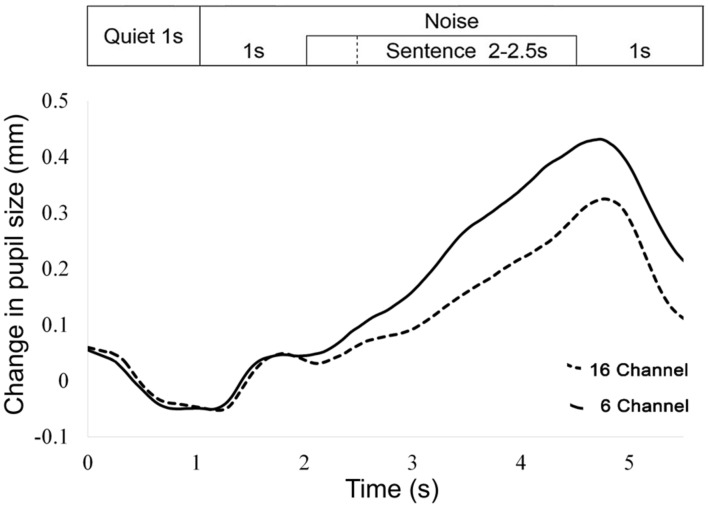
**Averaged pupil size over time for all trials and participants, for 16- and 6-channel vocoding.** The 1 s time-point refers to the beginning of noise. On this graph, all sentences finished at the 4.5 s time-point.

For each trial, the mean pupil size measured between 0 and 2 s was subtracted from the peak pupil size identified between 2 and 6 s (see **Figure 2** for an example of the pupil response during the experiment).

### Behavioral Measures

A behavioral test session was conducted with each participant to obtain a self-reported measure of effort during the sentence recognition task, which could be later compared to the physiological measures. This measure could not readily be obtained during the physiological test session because of the randomization of SNRs at each trial. The behavioral testing was performed in an acoustically treated room, with the equipment calibrated prior to each participant’s session. The speaker was positioned one meter from the participant at 0° azimuth. An adaptive procedure was chosen to obtain effort ratings at three SNRs around the mid-range of each participant’s performance-intensity function. The speech-in-noise algorithm and software used were developed by the National Acoustic Laboratories to obtain speech reception thresholds (SRT, the signal to noise ratio at which 50% of words were correctly perceived; see Keidser et al., 2013 for a comprehensive review). Target sentences were presented at 65 dB and the background noise was modulated using an adaptive procedure. The participant’s SRT was calculated when the standard error was less than 0.8 dB. The noise was then presented at a fixed level based on the participant’s SRT with 1 list (16 sentences), to validate the accuracy of the initial SRT calculation. Finally, the noise was fixed at -3 and +3 dB relative to their SRT and two lists per condition were presented, so that performance could be measured in easier and more difficult conditions. Thus, the conditions presented were: 50%SRT, 50%SRT(-3 dB), and 50%SRT(+3 dB) in the 16- and 6-channel vocoded conditions. All presentations were counterbalanced across participants for level and vocoding. After each presentation, participants were asked to rate the perceived effort invested in each SRT condition on a Borg CR10 scale (Borg, 1998).

### Statistical Methods

Linear mixed-effects models with a random intercept for individual were used for all analyses to control for repeated-measures over different levels of SNR on individuals. While random slopes were also of interest, these models failed to converge and were therefore not utilized.

Models for SRS were built by comparing a model with SNR, presentation mode and channel vocoding to a model containing SNR, presentation mode, channel vocoding and the interaction between SNR and presentation mode. The terms were fitted in the order described although no result difference was found if they were added to the model in a different order. Likelihood ratio tests were used to compare fixed effects of the simpler and more complex models after fitting the model using maximum likelihood. Where an interaction was not significant, the main effects model results were reported. All categorical variables used treatment contrasts (whereby all levels were compared with a reference level). *P*-values less than 0.05 were considered significant for all analyses.

Models for perceived effort, pupil size and alpha power were built by comparing a model with SNR and channel vocoding as main effects to a model with an interaction between SNR and channel vocoding. Because visual inspection of the change in pupil size and alpha power over SNRs suggested non-linear changes for one or both channels, models sequentially including a quadratic term for SNR (i.e., SNR^2^) and then a cubic term for SNR (i.e., SNR^3^) with an interaction between each term and vocoding channel were used to determine if the effects were similar for both channels. Again, likelihood ratio tests were used to compare models. These models are reported separately by channel vocoding (6 and 16) to aid interpretation. Models with a quadratic term are used to describe a simple curvilinear change while cubic terms are used to explain more complicated curvature with more than one change in the direction of the curve.

To account for the use of repeated measures on individuals, correlations presented in the results section are the average of the correlations calculated for each individual. Analyses were performed in R version using the nlme Package. This study was conducted under the ethical oversight of the Human Research Ethics Committee at Macquarie University (Ref: 5201100426).

## Results

### Performance-Intensity Functions and Effort Ratings

**FIGURE 3:**
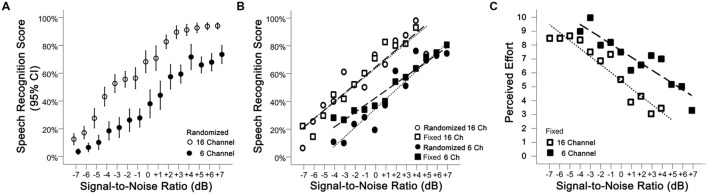
**(A)** Performance-intensity functions (mean plus 95% confidence intervals) are shown for the 16-channel (open circles) and 6-channel (closed circles) vocoded sentences measured during the physiological test session where SNRs were randomized. **(B)** Performance-intensity functions across the behavioral (squares) and physiological (circles) test sessions are very similar. **(C)** Mean effort ratings for 16-channel and 6-channel vocoded material measured in the behavioral test session.

Performance-intensity functions were measured during the behavioral test session (using 3 fixed SNRs per participant) and the physiological test session (using randomized SNRs across the 15 levels of noise). As seen in **Figure 3A**, SRSs measured during the physiological test session increased with SNR (*p* < 0.001) for both vocoding levels [16 ch: *r* = 0.93 (95% CI: 0.92 to 0.94); 6 ch: *r* = 0.92 (95% CI: 0.91 to 0.94)]. As expected, SRSs were significantly greater with the 16-channel material compared to the 6-channel (mean difference 26.72%, 95% CI: 22.12 to 31.31%, *p* < 0.001, **Table 1**). **Figure 3B** displays the performance-intensity functions where the three SNR levels presented in the behavioral session (fixed presentation) were matched to the same three SNRs measured during the objective session (randomized presentation). There was no evidence for a difference in the pattern of change in SRS between the fixed and random modes of presentation across the SNR levels, after adjusting for channel vocoding (*p* = 0.50, **Table 1**). For the 16-channel vocoding, for every unit increase in SNR, SRS increased by 6.44% (95% CI: 5.07 to 7.82%) for the fixed versus 6.47% (95% CI: 5.12 to 7.82%) for the randomized presentation, showing that the slopes by mode of presentation overlap considerably. Similarly, for the 6-channel vocoding, for every unit increase in SNR, SRS increased by 5.47 (95% CI: 4.29 to 6.64%) for the fixed versus 6.65% (95% CI: 5.13 to 8.18%) for the randomized presentation.

**Table 1:** Results for the linear mixed-effects models.

Coefficient	SRS	Perceived effort	Alpha power	Pupil size Linear	Pupil size Cubic
Intercept Slope (SE) *t*-value *p*-value	38.325 (2.110) 18.160 <0.001	7.506 (0.367) 20.414 <0.001	118.248 (16.382) 7.218 <0.001	0.401 (0.065) 6.174 <0.001	0.374 (0.067) 5.577 <0.001
SNR Slope (SE) *t*-value *p*-value	6.486 (0.453) 14.304 <0.001	-0.497 (0.068) -7.334 <0.001	0.0129 (1.242) 0.010 0.992	0.007 (0.003) 2.297 0.022	0.025 (0.008) 3.111 0.002
SNR^2^ Slope (SE) *t*-value *p*-value	–	–	–	–	0.001 (0.001) 1.739 0.083
SNR^3^ Slope (SE) *t*-value *p*-value	–	–	–	–	-0.001 (0.000) -2.390 0.018
Presentation Slope (SE) *t*-value *p*-value	1.509 (1.993) 0.757 0.450	–	–	–	–
Channel Slope (SE) *t*-value *p*-value	26.715 (2.327) 11.483 <0.001	-2.064 (0.321) 6.433 <0.001	21.719 (7.586) 2.863 0.004	-0.086 (0.019) -4.441 <0.001	-0.045 (0.029) -1.545 0.124
SNR*Presentation Slope (SE) *t*-value *p*-value	-0.383 (0.571) -0.672 0.503	–	–	–	–
SNR*Channel Slope (SE) *t*-value *p*-value	–	-0.105 (0.093) -1.128 0.263	-4.352 (1.756) -2.478 0.014	-0.016 (0.004) -3.470 <0.001	0.039 (0.011) -3.430 <0.001
SNR^2^*Channel Slope (SE) *t*-value *p*-value	–	–	–	–	-0.002 (0.001) -1.924 0.055
SNR^3^*Channel Slope (SE) *t*-value *p*-value	–	–	–	–	0.001 (0.000) 2.229 0.027

*Channel 6 was the reference level for channel; Random was the reference level for presentation mode. SRS, Speech Recognition Score; SNR, Signal-to-Noise-Ratio; SNR^*2*^, quadratic model; SNR^*3*^, cubic model.*

**Figure 3C** shows the mean effort ratings measured after each of the fixed SNR sentence blocks. There was no interaction between SNR and channel vocoding (*p* = 0.26, **Table 1**) indicating no evidence of a different pattern of effort over SNR between the two channels. Excluding the interaction term, LME regression confirmed that perceived effort averaged over channels significantly decreased (*p* < 0.001) with increasing SNR (-0.55, 95% CI: -0.65 to -0.45). SRS with 6-channel vocoding required on average 2.10 units more effort than 16-channel vocoding (95% CI: 1.47 to 2.74; *p* < 0.001).

### EEG Analyses

#### Effect of Vocoding on Baseline Alpha

A LME regression was used to examine the effect of vocoding (conducted during different test sessions) on alpha power during baseline. No significant difference was found between16- and 6-channel vocoding (mean difference = 0.69 mcV^2^, 95% CI: -1.47 to 2.85, *p* = 0.53). This suggests that overall, participants had similar alpha power baselines on both test sessions.

#### Alpha Power Change and SNR

**FIGURE 4:**
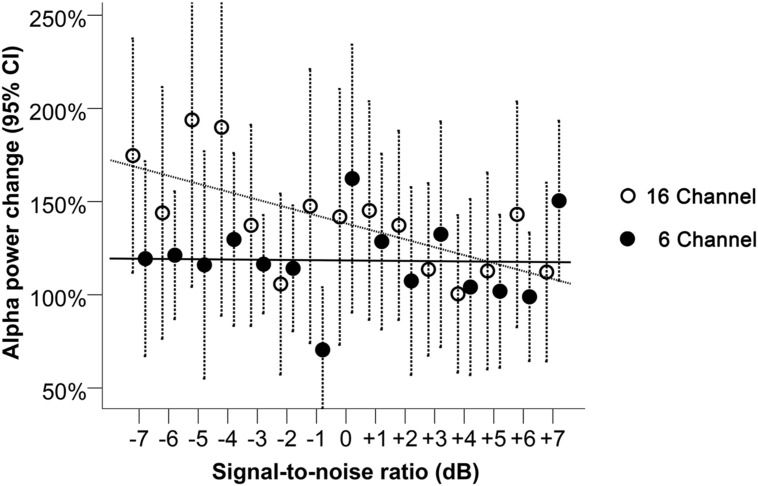
**Alpha power change relative to baseline during 16- and 6-channel vocoded sentence recognition at SNRs between -7 and +7 dB.** Dashed bars indicate 95% CI. The trend lines shown correspond to the best model fit, respectively, for the 16- and the 6-channel conditions.

Alpha power was processed as a relative change from baseline in noise, for each trial. A LME regression model suggested a significant interaction effect between SNR and channel vocoding on alpha power change (*p* = 0.01, **Table 1**). Specifically, for the 6-channel vocoding, there was no evidence of a change in alpha power over the different SNRs (0.01%, 95% CI: -2.38 to 2.41%); *p* = 0.99) while for the 16-channel vocoding, for every unit increase in SNR, alpha power decreased by 4.34% (95% CI: 1.94 to 6.73% decrease; *p* < 0.001). Non-linear models using a quadratic or cubic term for both channel vocoding did not improve model fit compared to a linear model (log likelihood -2632.18 vs. -2632.57, *p* = 0.68 and -2631.87 vs. -2632.57, p = 0.84, respectively). As seen in **Figure 4**, the largest separation between 16- and 6-channel vocoding was in the most challenging (lower) SNRs.

### Pupil Analyses

#### Pupil Size Change from Baseline

For the pupil size, a LME model was conducted to verify the effect of vocoding (conducted during different test sessions) on baseline, while controlling for repeated measures. The pupil size during baseline was found to be significantly larger during the second session [6-channel (harder condition); mean difference = 0.56 mm, 95% CI: 0.47 to 0.64 mm, *p* < 0.001].

#### Pupil Size Change and SNR

**FIGURE 5:**
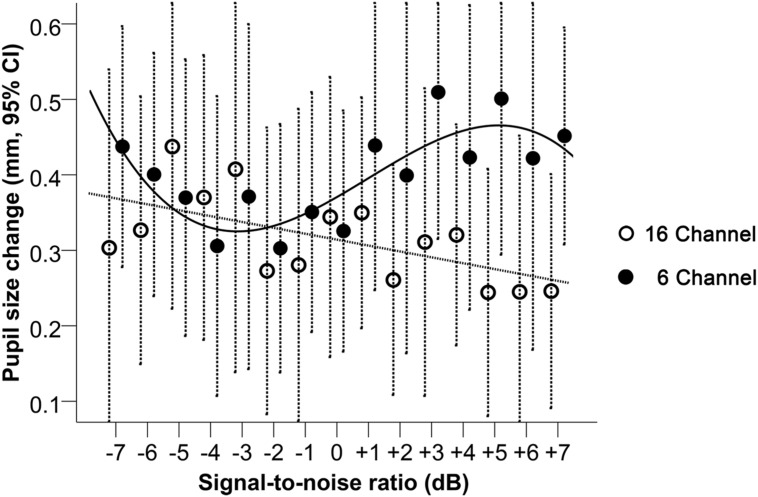
**Pupil size change relative to baseline during 16-and 6-channel vocoded sentence recognition at SNRs between -7 and +7 dB.** Dashed bars indicate 95% CI. The trend lines shown correspond to the best model fit, respectively, for the 16- and the 6-channel conditions.

Looking at the pupil size change relative to baseline (**Figure 5**), A LME regression model with only a linear term in SNR indicated a significant interaction effect between vocoding and SNR (*p* < 0.001, **Table 1**). For every unit increase in SNR, pupil size significantly increased by 0.007 mm (95% CI: 0.001 to 0.014 mm; *p* = 0.02) for the 6-channel vocoding while it significantly decreased for the 16-channel (mean change -0.008 mm, 95% CI: -0.015 to -0.002 mm; *p* = 0.01). Visual inspection of the relationship between pupil size and SNR indicated a potential non-linear relationship. As such a mixed effects model for pupil diameter containing a cubic term for SNR (**Table 1**) had significantly better fit compared to a linear model (log likelihood 97.6 versus 92.4, *p* = 0.04) or quadratic model (log likelihood 97.6 versus 94.4, *p* = 0.04). An interaction between the cubic term and channel was significant (*p* = 0.03). Examination of the relationship between pupil size and SNR within each channel indicated that with 16-channel vocoding, there was no significant effect of a quadratic term (*p* = 0.34) or cubic term in SNR (*p* = 0.46), while there was strong evidence of a cubic relationship (*p* = 0.01) for the 6-channel vocoding.

### Individual Alpha Power versus Pupil Size Change Comparisons

At the individual level, alpha power change was not found to be significantly correlated (*p* > 0.05) with pupil size change for either the 16-channel (mean *r* = 0.05, 95% CI: -0.16 to 0.26) or the 6-channel vocoding (mean *r* = -0.10, 95% CI: -0.35 to 0.16).

## Discussion

The results of this study suggest that, while there was a significant and expected difference in speech recognition performance and effort rating between the 6- and 16-channel vocoded material across the 15 SNRs, the mean changes observed in the physiological measures (alpha power and pupil size) were less predictable. Significant relationships were found between mean pupil dilation and SNR, and mean alpha power and SNR for 16-channel vocoded sentences, showing a similar trajectory of change; i.e., larger pupil responses and larger alpha power change were measured for less intelligible speech. For the pupil response only, there was also a significant non-linear relationship with SNR with the 6-channel vocoded sentences, whereby pupil dilation was larger in the hardest and easier conditions. This is perhaps consistent with the non-linear change in pupil dilation with changes in task difficulty that have been shown previously (Granholm et al., 1996; Zekveld and Kramer, 2014). Further, significant interactions between SNR and vocoding were seen in both physiological measures, although the largest difference between alpha power change was observed in the least intelligible conditions (more negative SNRs) whereas the largest difference in the pupil dilation was observed in the most intelligible conditions (more positive SNRs).

The linear association between SNR and pupil dilation for the 16-channel vocoded sentences, and the comparatively larger pupil dilation for the 6-channel compared with the 16-channel vocoded sentences at more positive SNRs (≥+2 dB), is similar to that observed in previous studies, i.e., larger pupil size is observed with greater cognitive load (Kahneman and Beatty, 1966; Granholm et al., 1996; Winn et al., 2015). Larger pupil dilation relative to baseline is typically measured during more cognitively demanding speech processing tasks. For example, poorer SNRs (Zekveld et al., 2010), greater spectral degradation with channel vocoding (Winn et al., 2015), single-talker compared with noise maskers (Koelewijn et al., 2012), randomized SNRs compared with fixed SNRs (Zekveld and Kramer, 2014), grammatical complexity (Schluroff, 1982) or perceptual effort with hearing loss (Kramer et al., 1997). Certainly the results of the current study support an increase in pupil dilation for the most challenging SNRs with the 16-channel vocoded sentences. However, the relationship between pupil dilation and SNR for the 6-channel vocoded sentences in the current study was not simple, where the mean pupil dilation across subjects plateaued for moderately negative SNRs and showed an increase with increasing speech intelligibility. It is possible that the changes in the pupil size across the 15 SNRs for the 6-channel vocoded sentences could reflect the non-linear behavior of the pupil size that has been observed when task difficulty exceeds capacity (Peavler, 1974; Granholm et al., 1996; Zekveld and Kramer, 2014). For example, it has been demonstrated that pupil dilation systematically increases with task difficulty (such as with a digit recall task), until it reaches or exceeds the limits of available cognitive resources, whereby it either asymptotes (Peavler, 1974), declines (Granholm et al., 1996), or shows both a decline followed by an asymptote for the most challenging intelligibility conditions (Zekveld and Kramer, 2014). An alternative explanation is that the noise levels *per se* could have influenced pupil dilation at the more negative SNRs (noise levels reached a maximum of 72 dB), where mean pupil dilation for both 16- and 6-channel vocoded sentences was similar. While Zekveld and Kramer (2014) attempted to reduce the likelihood of noise affecting pupil dilation by controlling the overall signal level while changing the SNR, in the current study, a fixed signal level was used with modulated levels of noise. Pupil dilation has been shown to be modulated by acute stress (Valentino and Van Bockstaele, 2008; Laeng et al., 2012) and animal studies have demonstrated that long-term effects of non-traumatic noise is associated with increased cortisol levels, hypertension and reduced cardiovascular function (see Gourévitch et al., 2014 for a review). A recent study looking at physiological measures of stress during listening in noise found that adults with hearing loss, who are constantly exposed to degraded speech, had higher autonomic system reactivity compared to adults with normal hearing, at similar performance levels (Mackersie et al., 2015). Therefore, while the noise levels in the current study were short-term, this may have caused a phasic stress reaction which could have influenced pupil dilation. This hypothesis, however, is not supported by studies suggesting that the pupil dilates with negative affect (Partala and Surakka, 2003).

The change in mean alpha power, relative to baseline, showed an enhancement of alpha activity in both 16-channel and 6-channel vocoding conditions, consistent with the inhibition hypothesis, where activity that is not related to the goal-directed task is actively inhibited (Klimesch et al., 2007). Therefore, it has been suggested that alpha enhancement which occurs during a speech-in-noise task results from the enhancement of auditory attention through the active suppression of noise (Strauß et al., 2014). However, most studies assessing alpha power change with vocoded speech material (Obleser and Weisz, 2012; Becker et al., 2013; Strauß et al., 2014) or during the processing of semantic information (Klimesch et al., 1997) have shown a reduction of alpha power, which is consistent with active cognitive processing of speech information. Specifically, the results of the current study appear contradictory to those reported by Obleser and Weisz (2012) using noise vocoded (2-, 4-, 8-, and 16-channels) mono- bi- and tri-syllabic words. They showed less alpha power suppression, of posterior-central alpha power with decreasing intelligibility measured between 800 and 900 ms post word onset. However, the task across the two studies was not the same. In the current study, participants were required to repeat the vocoded sentences, whereas in the Obleser and Weisz (2012) study, participants were asked to rank the comprehension of vocoded words without attending to the linguistic or acoustic aspects of the speech materials. While previous studies have shown a very high correlation between SRSs and rating scores, it is unclear whether the pattern of event-related oscillatory cortical activity measured during these different tasks is the same. Further, the types of analyses conducted across studies are not the same. For example, while Becker et al. (2013) demonstrated that mean alpha power during the region of interest (ROI) between 480 and 620 ms is reduced as speech intelligibility is increased (using monosyllabic French words), this was an absolute measure of alpha power rather than a change relative to the baseline. Variability of whether alpha power was increased or decreased was observed within studies. For example, Becker et al. (2013) showed the mean trajectory of change in alpha power during noise-vocoded monosyllabic words and demonstrated that alpha power is enhanced in the less intelligible conditions (similar to our results) but is suppressed in the most intelligible conditions (similar to the results shown by Obleser and Weisz, 2012). Further, using an auditory lexical decision task, Strauß et al. (2014) demonstrated mean increases of alpha power occurred for clear pseudo-words but a reduction was observed for ambiguous and real-words, which parametrically changed as the clarity of the words increased. Finally, using 18 younger and 20 older healthy adults, Wöstmann et al. (2015) demonstrated that decreases in mean alpha power which occurred as speech intelligibility increased (using four syllable digits masked by a single speaker) appeared to be driven by the older adults rather than an effect across the entire population. Given the differences in the types of speech stimuli used across the different studies, the task required, as well as the ROI used to assess alpha power changes (i.e., during or after the speech tokens), and the different populations assessed (older versus younger adults), further investigation of alpha power is needed to better understand the changes observed and how this might be used as an objective measure of attentional effort and/or cognitive load for the individual.

Within the current study, while a significant interaction was found between 6- and 16-channel vocoding for both alpha power and pupil size change, the trend patterns differed. The magnitude of the difference between both vocoding levels was greater in the most challenging SNRs for alpha power, but in the least challenging SNRs for the pupil size. This could suggest that these physiological responses are driven by different neurophysiological or attentional networks (Corbetta and Shulman, 2002; Corbetta et al., 2008; Petersen and Posner, 2012). There is a vast literature on attentional effort which suggests that discrete neuroanatomical areas encode specific cognitive operations (“processors”) that are involved in attention, which are modified by “controllers” depending on the type of attentional tasks required (see Power and Petersen, 2013). While the majority of the literature in this field focuses on the visual modality, there is evidence to suggest that similar processes should be evident when listening to degraded speech, such as listening in noise (Spagna et al., 2015). The main determinants of attentional allocation would then be; the identification of the appropriate processing strategy needed to undertake the speech perception task, the maintenance of attention during the task, and the processing of errors to increase (or, at least, reduce declines in) performance. Further, these processes may work synergistically under less cognitively demanding conditions but diverge under more challenging conditions, or conditions which have different types of attentional requirements (Vossel et al., 2014). It is also possible that different processors and controllers are used by different individuals to undertake these cognitively demanding task, which may have led to a lack of correlation between alpha power change and pupil dilation change within individuals. Corbetta and Shulman (2002) proposed the existence of two anatomically distinct attention networks; the dorsal fronto-parietal network, which is involved in the top–down voluntary or goal-directed allocation of attention (which includes preparatory attention and orienting within memory), and the ventral fronto-parietal network, which is involved in the involuntary shifts in attention. It is proposed that under normal circumstances, the ventral network is suppressed but is activated by unexpected, novel, salient, or behaviorally relevant events. Where this occurs, it is assumed that a “circuit-breaking” signal is sent to the dorsal attention network, resulting in reorienting, or shifting in attention toward this new event (Corbetta et al., 2008). It has been proposed that the locus coeruleus-norepinephrine (LC-NE) system modulates the functional integration of the entire cortical attentional system (Corbetta et al., 2008; Sara, 2009), whereby NE released by the LC triggers the ventral network to interrupt the dorsal attention network (Bouret and Sara, 2005) and reset attention. This ensures a coordinated rapid and adaptive neurophysiological response to spontaneous or conditioned behavioral imperatives (Sara and Bouret, 2012).

Pupil dilation is under the control of the LC-NE system, therefore it may be reasonable to assume that indirect attention tasks may be associated with the changes in pupil dilation observed in the current study. It has been proposed that pupil dilation is modulated by both staying on task and choosing between alternatives (exploration; Aston-Jones and Cohen, 2005). Therefore, a complex task, such as the perception and comprehension of a moderately intelligible (vocoded) speech signal, may result in changes in pupil dilation that reflect the interaction between different processing strategies. Alpha power changes have been associated with top–down inhibition of task irrelevant brain regions, and it has been suggested that alpha power is under the control of the dorsal attention network (Zumer et al., 2014). Further, increases in alpha power may inhibit the ventral attention network, preventing reorienting to irrelevant stimuli during goal-directed cognitive behavior (Benedek et al., 2014). While other models of attention exist (Seeley et al., 2007; Petersen and Posner, 2012), it is clear that a simple association between a physiological measure of attentional effort and task difficulty (e.g., changes in speech intelligibility) fails to consider the multiple autonomic cognitive operations as well as the voluntary control of attention that reflects effortful cognitive control (see Sarter et al., 2006). It is recognized that there is a dynamic interplay between the bottom–up sensory information and the top–down cognitively controlled factors (which may be either under automatic or voluntary control), such as knowledge, expectations and goals, that can be modulated by motivational factors, such as payment for participations (Tomporowski and Tinsley, 1996) and genetic influencers (Fan et al., 2003). Therefore, it is reasonable to assume that considerable variability in attentional allocation could exist between individuals undertaking a highly complex task.

An alternative explanation is that the within-subject variability of sustaining on-task attention toward sentences with unpredictable levels of intelligibility, was greater under the more challenging noise vocoding conditions (6-channel) where the effort-reward balance was not as high compared with the 16-channel vocoded materials. Sustaining attention on a complex task is challenging (Warm et al., 2008) and requires suppression of internal tendencies of mind-wandering, a default network activation that typically occurs during low task demands (Christoff et al., 2009; Gruberger et al., 2011), with concomitant activation of the goal-directed dorsal fronto-parietal attentional network (Corbetta and Shulman, 2002). Fluctuations in sustained attention can occur with stress, distraction with competing stimuli, fatigue, or lack of motivation toward the task, and are commonly associated with a decline in performance (Hancock, 1989; Esterman et al., 2012). As stated by Esterman et al. (2012) “as the neural systems supporting task performance appear to shift with one’s attentional state, failure to account for attentional fluctuations may obscure meaningful information about underlying mechanisms”. Certainly, some people have a preponderance to mind-wandering (Mason et al., 2007). This may be a confounder to the results of the current study comparing physiological responses to a range of SNRs, despite the ecological validity that this may have to their ability to follow conversations within multi-talker environments. That is, the variability in the physiological measures may, in fact, provide important information about the individual’s processing of degraded speech that is not captured within more common behavioral measures of speech perception. For example, a recent study by Kuchinsky et al. (2016), suggests that individual differences in the pupillary response of older adults with hearing loss during a monosyllabic word recognition task was related to task vigilance (less variability in response time) and to the extent of primary auditory cortical activity. Therefore, pupil dilation may index the magnitude of the engagement between bottom–up sensory and top–down cortical processing which is increased with greater degradation of the speech signal (influenced by poorer SNR, reduced spectral information, or hearing loss).

Significant differences in the baseline data were also observed between the 6- and 16-channel vocoding for pupil size, but not for alpha power. These two levels of vocoding were assessed during different sessions for all subjects, therefore this could be due either to a session effect, or to a difference in the level of cognitive effort that was maintained throughout the session. Given that the results are consistent with an increase in cognitive load during the 6-channel vocoded session, it is likely that the difference in the tonic pupillary response across the two physiological measures sessions (16- versus 6-channel vocoded-sentence tasks) resulted from differences in vigilance or the awareness of errors in performance during the more cognitively challenging task (Critchley, 2005; Ullsperger et al., 2010).

Limitations of the study include the relatively small number of participants included in the final data analysis (particularly for pupillary measures), and that only 16 sentences were presented for each SNR level (scored as 50 words across the set of 16 sentences) in each condition, reducing statistical power. Further, the test set-up restricted people from responding normally to an effortful task (i.e., a number of participants tended to close their eyes during the stimuli presentation but were instructed to keep their eyes opened). Explicitly investing effort in trying to keep their eyes opened despite the natural tendency to want to close them may have in itself created changes in pupil size and alpha oscillations. This may also have added an additional stressful component to the task.

## Conclusion

The results of this study suggest that the relationship between task difficulty and both pupil dilation and alpha power change was similar for the 16-channel vocoded sentences (high intelligibility), which might suggest that the attentional networks are operating with high concordance, or in a consistent and predictable manner across the SNRs. However, further degradations in the speech intelligibility, using the 6-channel vocoded materials, could have produced a discordant relationship between the attention networks, or different processors (such as linguistic strategies) may have been used to comprehend the speech signal. Importantly, however, given the considerable interest in assessing listening effort within clinical settings (see McGarrigle et al., 2014), it is important to ensure that we have a solid understanding of what these physiological measures are assessing, and how to interpret the responses for the individual. Certainly, the results of this study do not currently support the clinical use of these physiological techniques as sufficiently sensitive to provide complementary information about listening effort to existing measures of speech perception performance. To be clinically viable in a hearing rehabilitation setting, such objective indices of effort should be more sensitive to changes in auditory input than existing measures of speech perception performance or subjective ratings of effort. The behavior of these indices should also be predictable across a range of performances and speech degradation to be applicable to the range of hearing loss and devices available, including hearing aids, and cochlear implants.

## Author Contributions

Original idea: PL, CM, IB. Protocol development: PL, IB, CM, RI. Data collection: CYL, LG, PL, IB. Writing of manuscript: CM, IB, LG, KM, CYL. Data processing and analyses: RI, PL, PG, IB, CM, KM.

## Conflict of Interest Statement

The authors declare that the research was conducted in the absence of any commercial or financial relationships that could be construed as a potential conflict of interest.
